# Supplementary material for: Patient-Centric Medicine Design: Key Characteristics of Oral Solid Dosage Forms that Improve Adherence and Acceptance in Older People
Source: Pharmaceutics. 2020 Sep 23;12(10):905. doi: 10.3390/pharmaceutics12100905 (PMC7598259; doi:10.3390/pharmaceutics12100905)
Supplement: Supplementary file 1 [file pharmaceutics-12-00905-s001.pdf]

pharmaceutics

Supplementary Materials: Patient-Centric Medicine Design: Key Characteristics of Oral Solid Dosage Forms that Improve Adherence and Acceptance in Older People

Zakia Shariff, Daniel Kirby, Shahrzad Missaghi, Ali Rajabi-Siahboomi and Ian Maidment

Table S1. Participant characteristics.

(**a**) Older People.

Code	M/F	Age	Ethnicity
P1	M	69	A
P2	M	66	A
P3	F	71	A
P4	F	80	A
P5	F	81	A
P6	F	66	A
P7	M	74	A
P8	F	69	J
P9	F	81	A
P10	F	74	A
P11	M	77	A
P12	F	97	A
P13	F	94	A
P14	M	72	A
P15	M	92	A
P16	M	93	A
P17	M	94	A
P18	F	67	A

(**b**) Informal Carers.

Code	M/F	Age	Ethnicity
C1	F	69	A
C2	F	64	A
C3	F	48	A
C4	F	61	A
C5	F	63	M
C6	M	51	J
C7	F	70	A

(**c**) Health and Social Care Professionals.

Code	M/F	Role	Period of time in Current role
HCP1	M	GP	6 years
HCP2	F	Health Care Assistant	14 months
HCP3	M	Trainee GP	7 months
HCP4	F	Consultant Pharmacist	16 years
HCP5	M	Prescribing Advising Pharmacist	4 years
HCP6	F	Clinical Lead Pharmacist/Practice based pharmacist	3 years/15 years
HCP7	M	Specialist in Pharmaceutical Public Health	17 years
HCP8	F	Older Person's Specialist Pharmacist	9 years
HC9	F	General Adult Nurse	4 months
HC10	F	Chief Nurse	3 years
HC11	F	Nurse for Safeguarding in Adults	7 years
HCP12	F	Clinical Nurse Specialist/ Continence Service Manger	23 years
HCP13	F	Practice based Pharmacist	1 year
HCP14	F	Community Pharmacist	8 years
HCP15	F	Medicines Management Technician	6 months
HCP16	F	Pharmacy Technician	2 years
HCP17	F	Hospital Staff Nurse	15 years
HCP18	F	Deputy Ward Manager	5 years
HCP19	F	Locum Senior House Officer (Non-Consultant Hospital Doctor)	5 weeks
HCP20	F	Staff Nurse at Nursing Home	3 years
HCP21	F	Senior Care Support Worker	10 years
HCP22	M	Care Support Worker	3 years
HCP23	M	Care Support Worker	3 years
HCP24	F	Senior Care Worker	5 years
HCP25	F	GP	10 years
HCP26	F	Clinical Lead at Nursing Home	7 months
HCP27	F	Care Team Leader at Nursing Home	2 years

Key
A: White British
J: Asian/Asian British: Pakistani
M: Black/Black British: Caribbean

Table S2. Illustrative data extracts in for preparations that are difficult to swallow due to the size and shape.

Medication	Illustrative Data Extract
Metformin	I think if it (metformin) was any bigger it might be a bit difficult (to swallow) because it is fairly big. It's bigger than any of the ones that you've got there (P1)
	These are big buggers, these Glucophage ones… And they're dry, and they would go down a lot easier with a coating or something I would think, and maybe you know, a pillow or whatever shape (P7) *
	Well the metformin are more difficult because they're quite a big round tablet, I mean they're much bigger than the ones you've got here. So they're circular like that (10 mm round), but quite a bit bigger and thicker. So that's more difficult. (C3)
	You have to drink a lot, you have to make sure you know, you drink before, during and after really. (C4) (In reference to Glucophage)
	I mean if you get up to 1g tablets then they're a, there's a larger group of people that can't take them so, you know, your metformin 1g for example would be, there'll be a group of people who are on 500mg, two tablets, because they just simply can't get the pill down so (HCP1)
	If like for example, we come across patients on metformin, they're big tablets, they say they get GI problems so we give them a slow-release preparation, then as we titrate the dose and put them on higher strengths, they end up being like horse pills and the patients are like 'it's not that I don't want to take them, it's just I can't swallow them' or you know, like 'I have to psyche myself up because of the gag reflex' (HCP6)
	If they could all, I don't know how you would do this but I know that the size puts a lot of people off, so if they could compact like a, like whatever that is, I mean that (18 × 7 mm caplet) looks like the kind of shape a metformin would be. I don't know like how you'd be able to make a metformin that sort of size (6mm round)? (HCP15)

Paracetamol	Yesterday I was trying and I thought I'm sick of not being able to swallow them (paracetamol), and do you know I can put one in the back of me mouth and I've two glasses of water and it's still there, it never goes down. (P4)
	I much prefer the caplets of paracetamol. I do break them in half but I do find them very much easier to swallow than, I always, on the odd occasion that I've taken the round paracetamol, I think those are quite difficult to swallow to be quite honest. (P5) *
	I think these ones (caplet) they are a bit easy to take, but the other round ones sometimes it's hard to swallow them and I have to sip a lot of water with it (P8) *
	Paracetamol I find is very large… (I break it) in two, two I can just about get down but it's a bit of a swallow (P10)
	Although I do have trouble with paracetamol. Unless that goes in straight... I find it very hard to swallow (P11)
	So occasionally it (paracetamol) seems as if it might be getting stuck (P18)
	They're (paracetamol) like that (18 × 7 mm caplet) but they're like a caplet, a coated one, but that shape, perhaps a little bit deeper than that. He doesn't find them easy to swallow at all. And really weird just getting them down him (C2)
	And the paracetamol are similar to these ones, (18 × 7 mm caplet) they're relatively long, and fairly thin, but they're still harder than for example the Simvastatin that's more that shape (12 × 7 mm oval) (C3)
	Paracetamol, is that (bulkiness) just because of the amount of active ingredient, is that? … Because that's the one medication usually patients complain about. (HCP3)
	We can see some of the, some paracetamol in this shape isn't it, so people, usually they'll ask us to break the caplet. The caplets, usually elders ask to break it in two so they can make it more easier. (HCP17)
	Sometimes they are big and very round, like round paracetamol, they are quite difficult as well. (HCP18)
	If we changed, because some of them, if we changed the size, like, paracetamol's quite big, instead of changing the size, the best thing is just to like to prescribe in soluble form. (HCP20)
	Yeah. There have been a few instances where for example some paracetamol tablets, they prefer the caplet sometimes because it's easier to swallow (HCP25) *
Co-codamol	I go for the sort of bullet shape rather than the round ones. But, I mean, these are small enough for it (the shape) not to matter, but when you get into bigger pills, it's easier, things like the co-… Solpadol, yeah. (P3) *
	What you haven't got here, which I was expecting you to have is the thick round one. That type of paracetamol. Or the co-codamol which I take. And it's round and it's thick. And I struggle with that, it can make me feel like vomiting (C2)

Calcium & Vitamin D (including Adcal)	Swallowing is a bit difficult because they are quite a big tablet so I don't have to break it, but I have to swallow it with a lot of water (P8) (In reference to 16.5 mm × 8.5 mm oval calcium and vitamin D tablets).
	The Adcal are a bit sizeable (P9)
	Many times we'd see that a lot of patients would just not take their calcium, vitamin D tablets, they're large tablets (HCP14)
	On a, probably the only stuff that we've had complaints about are Adcal and metformin, I think is Adcal massive, it's a gram isn't it, so? (HCP1)
	So do I chew it? Do I dissolve it in water? This is a massive tablet and we worked out that it was a, I think it was an Adcal-D3 or something like that, calcium and vitamin D which he was to chew (HCP7)
	I mean Adcal-D3 is a really good example of one that it's very poor adherence because they don't like the size of it and they don't like the taste of it (HCP8)
Antibiotics	I mean if you were looking at azithromycin 500mgs, they're massive aren't they? And you might think "oh they're bloody big tablets so let's think about either the formulation or another macrolide maybe" (HCP7)
	I think it's whether they're going to be able, because there are certain antibiotics capsules which are really big, you know, for example metronidazole and flucloxacillin, even I at times have struggled, you know, to sort of take those and I think God if I've struggled then, you know, an older person, you know, some older people probably would really struggle with trying to swallow some of those (HCP9)
	I'd probably go that one (16.5 mm × 8.9 mm oval). [Shows interviewer]. Because that's probably about the size of a co-amoxiclav and a lot of patients can't take them ones because they say they're too big (HCP16)
	Yeah, we always face that issue with antibiotics. Whenever they prescribe antibiotics here it's always a problem because it's always big. (HCP24)
	I know antibiotics are and they're generally big. (HCP26)

* Indicates quotes illustrating relationship between size and shape.
